# Finished Genome Sequence of Bacillus cereus Strain 03BB87, a Clinical Isolate with B. anthracis Virulence Genes

**DOI:** 10.1128/genomeA.01446-14

**Published:** 2015-01-15

**Authors:** Shannon L. Johnson, Timothy D. Minogue, Hazuki Teshima, Karen W. Davenport, April A. Shea, Haven L. Miner, Mark J. Wolcott, Patrick S. G. Chain

**Affiliations:** a Los Alamos National Laboratory (LANL), BioScience Division, Los Alamos, New Mexico, USA; b U.S. Army Medical Research Institute of Infectious Diseases (USAMRIID), Diagnostic Systems Division (DSD), Frederick, Maryland, USA

## Abstract

Bacillus cereus strain 03BB87, a blood culture isolate, originated in a 56-year-old male muller operator with a fatal case of pneumonia in 2003. Here we present the finished genome sequence of that pathogen, including a 5.46-Mb chromosome and two plasmids (209 and 52 Kb, respectively).

## GENOME ANNOUNCEMENT

Bacillus cereus strain 03BB87 was isolated in 2003 from a 56-year-old male muller operator in Lubbock, Texas, USA, who perished from bacterial pneumonia (1). The strain carries a pX01 analogue plasmid (called pBCX01) that contains anthrax-associated virulence genes as well as a second, smaller plasmid. A copy of the organism was obtained from the Department of Defense Unified Culture Collection maintained at the U.S. Army Medical Institute for Infectious Diseases (USAMRIID) as BACI233.

Genomic sequencing included long-insert paired-end Roche 454 data (11× genome coverage), 100-bp Illumina standard data (283× genome coverage), and PacBio data (29× genome coverage). Each data set was assembled separately in a native assembler (Newbler, Velvet [2], or AllPaths [3]), and then the assemblies were combined using PhredPhrap (4, 5). Misassemblies were manually curated and addressed using in-house scripts and final manual corrections were made in Consed (6). The final assembly was annotated in-house using an Ergatis-based workflow manager (7). Data files are available in NCBI under accession number SRP049106.

The finished genome of B. cereus 03BB87 is 5,718,948 bp with a G+C content of 35.2%. The genome contains 5,788 protein-coding sequences, 107 tRNA genes, and 39 rRNA genes. A total of 74.3% of open reading frames (4,409) are protein-coding genes with function prediction. Preliminary review of the annotated genome finds resistance to tetracycline, vancomycin, and other antimicrobials.

### Nucleotide sequence accession numbers.

The genome is available in GenBank, with the chromosome listed as CP009941, pBCX01 as CP009940, and pBCN as CP009939.
